# Development and measurement of elbow and knee joints using an electro-goniometer in healthy subjects: A preliminary study

**DOI:** 10.1051/sicotj/2026016

**Published:** 2026-05-05

**Authors:** Sasipa Buranapuntalug, Nuchrada Chaitrakul, Pollapat Liamtragoolpanich, Chusak Thanawattano, Chatchai Buekban, Kornanong Yuenyongchaiwat

**Affiliations:** 1 Physiotherapy Department, Faculty of Allied Health Sciences, Thammasat University Pathumthani Thailand; 2 Thammasat University Research Unit for Physical Therapy in Respiratory and Cardiovascular Systems, Thammasat University Pathumthani Thailand; 3 Biomedical Electronics and Systems Research Team, Assistive Technology and Medical Devices Research Group, National Electronics and Computer Technology Center (NECTEC) 12120 Pathum Thani Thailand

**Keywords:** Range of Motion, Goniometer, Movement, Electronic Goniometer

## Abstract

*Introduction*: Range of Motion (ROM) assessment is a critical baseline metric for diagnosis, treatment monitoring, and rehabilitation goal setting. It significantly impacts patient well-being, aligning with Sustainable Development Goal 3 (SDG 3). However, the universal goniometer (UG), presents limitations regarding accuracy and practical efficiency in clinical settings. Therefore, this study aimed to determine the concurrent validity of an electronic goniometer named Goniwear compared to the UG for measuring elbow and knee angles. *Methods*: The validity of Goniwear involved 40 healthy volunteers stratified by age (20–39 and 40–59 years) and sex. Simultaneous active and passive ROM measurements were conducted three times using both UG and on flexion and extension of the elbow and knee joints. Data were analyzed using the intraclass correlation coefficients (ICC), which were calculated using a two-way random-effects model, and the Bland-Altman method was used to determine the limits of agreement (LoA) between the UG and Goniwear. *Results*: Reliability between the two instruments ranged from poor to excellent, depending on the joint and movement type. Elbow flexion and extension demonstrated consistently good to excellent reliability in both active and passive conditions (ICC = 0.84–0.91), with minimal bias and relatively narrow LoA. Knee flexion and extension showed poor to moderate reliability (ICC = 0.44–0.55), particularly for extension, accompanied by a wide LoA. Conclusion: Agreement between the UG and Goniwear varies across joints and movement conditions. While the instruments appear interchangeable for elbow movements, caution is warranted when interpreting knee ROM due to greater measurement variability. *Discussion*: The Goniwear demonstrates high validity for single-axis joints with fixed pivot points, suggesting strong potential for clinical application in specific contexts. *Trial Registration*: The Thai Clinical Trials Registry is TCTR20251120001.

## Introduction

Measurement of joint range of motion (ROM) is a fundamental component of musculoskeletal assessment and is widely used for diagnostic purposes, treatment planning, and evaluation of therapeutic outcomes. The universal goniometer (UG) remains the most commonly utilized instrument for quantifying joint motion in angular degrees. However, accurate ROM measurement using a UG is highly dependent on the examiner’s skill, experience, and proper identification of anatomical landmarks. The gold standard for measuring ROM is radiography; however, the harmful effects of radiography and the failure to repeat the test are also concerns [1]. Variability in instrument alignment, angle interpretation, and data recording may introduce examiner-related error, potentially compromising measurement reliability and validity [2].

To address the limitations associated with the UG, several studies have developed alternative devices for measuring joint ROM, including sensor-based technologies. Correll et al. (2018) developed a system that combined an accelerometer and a magnetic sensor to quantify shoulder joint ROM and compared its performance with that of a UG [3]. Their findings demonstrated good intra-rater reliability and acceptable validity [3]. Another study developed a three-dimensional global coordinate system–based sensor to measure angular displacement by comparing two spatial positions and comparing it with the UG [2]. The results demonstrated almost perfect reliability across all directions of knee movement. Validity was found to be very high for both active and passive knee flexion and high to moderate for both active and passive knee extension [2]. However, individual body composition factors, such as muscle mass, may influence the accuracy of angular measurements [4]. Moreover, despite these technological advancements, the devices do not fully overcome certain limitations, such as the inability to perform self-measurement independently. In addition, a smartphone with a real-time dynamic ROM might be beneficial for investigating rhythms of dynamic movement.

In contrast, electro-goniometers address several limitations of manual measurement by enabling continuous assessment of dynamic movement, thereby providing more objective and time-resolved data. In particular, inertial measurement units (IMUs), as a more advanced technology, enable wireless three-dimensional joint angle measurement without the need for precise alignment of the rotational axis [5]. This characteristic makes IMU-based systems especially suitable for capturing complex movements in real-world environments outside the laboratory setting [6]. However, traditional potentiometer-based electro-goniometers remain limited by measurement inaccuracies associated with skin movement artifacts and the requirement for accurate mechanical axis alignment [7, 8]. Similarly, IMU-based systems are subject to signal drift and cumulative error during prolonged continuous measurements, which may compromise long-term accuracy. Therefore, the study aimed to develop an electro-goniometer, a dynamic wearable ROM measuring device for the movement of the elbow and knee joints.

## Material and methods

**Figure 1:**
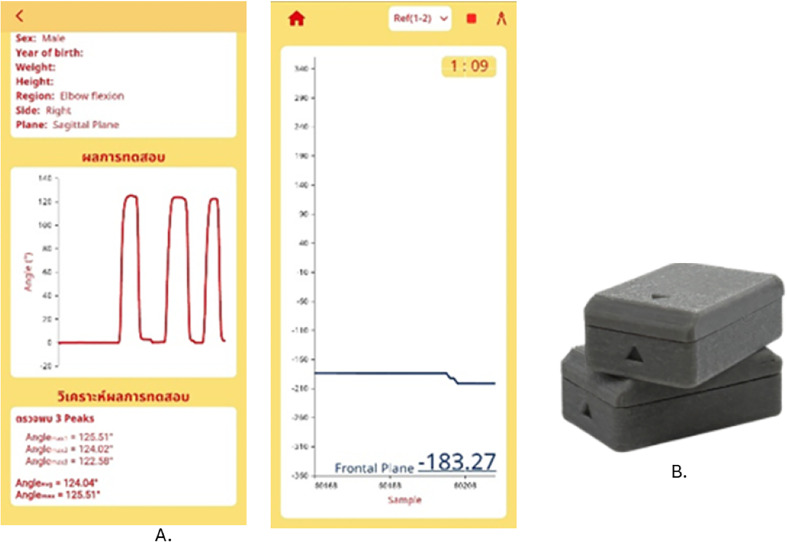
Goniwear.

The study developed the electro-goniometer, named Goniwear. The Goniwear is a portable, skin-mounted, two-module wearable system designed to estimate a human joint angle by measuring the relative orientation of two body segments adjacent to the joint. Each module is placed on opposite sides of the joint (e.g., proximal and distal segments) using double-sided adhesive tape to provide consistent skin contact during motion. During measurement, each module acquires raw inertial signals (accelerometer and gyroscope) from an onboard IMU and transmits the raw data to a mobile application via Bluetooth Low Energy (BLE). The mobile application estimates roll and pitch for each module and computes the joint angle by subtracting the orientations of the two modules after an initial calibration step that sets the relative angle difference to zero. The width, height, and length are 2.4 cm, 3.6 cm, and 1.5 cm. The smartphone application is operated by an Android application and displays real-time movement, showing a record number and a graph (Figure 1).

Each wearable module is implemented using an Arduino Nano 33 BLE board, which integrates a 9-axis IMU and BLE capability. In this study, only accelerometer signals are used to estimate roll and pitch, while yaw is excluded from the computation pipeline.

To measure a joint angle, Module A is attached to the segment on one side of the joint and Module B to the segment on the other side.

Mobile Application (Flutter/Android): Roll/Pitch Estimation and Joint Angle Computation. The Flutter mobile app acts as the central processing unit. It connects to both wearable modules over BLE, receives raw data streams, computes roll/pitch for each module, and outputs the joint angle in real time. The resulting relative angles are displayed in the mobile app as a real-time time-series plot, updated continuously so the user can observe joint movement dynamics immediately while the motion is occurring.

We selected the elbow and knee joints for assessment based on both biomechanical and sensor-technology considerations. The movements of these joints, particularly flexion–extension, occur predominantly within the sagittal plane, which is well suited for IMUs based angle detection. In addition, it can reliably detect angular displacement primarily within the sagittal plane through accelerometer and gyroscope data. Restricting measurements to primarily single-plane motion minimizes cross-axis interference and reduces computational complexity in sensor data processing, thereby improving measurement accuracy and validity. These joints are also commonly assessed in clinical practice, further supporting their selection for device validation.

To evaluate the validity of the UG and Goniwear, 40 participants aged 20–59 years, both males and females with a normal body mass index (BMI: 18.5–24.9 kg/m^2^) were included. Participants presenting with open skin wounds, infected lesions, or known hypersensitivity to transparent adhesive dressings were excluded. A total of 40 participants were enrolled and stratified by age into two groups: 20–39 years and 40–59 years. Each age group included 20 participants and was further stratified by sex, with equal representation of males and females. As this study was designed solely to determine the validity of a prototype, Goniwear in a volunteer population, the device was used to detect joint ROM via embedded sensors and to convert the data into numerical values and graphical outputs. Accordingly, the sample size for this study was determined using Item Calibration and Pearson Correlation Estimates under the Rasch measurement model to achieve a 95% confidence interval [9]. The circumference of the upper arm, forearm, thigh, and lower leg was measured at the point of maximal girth on the dominant side while the participant was in a standing position. Anatomical reference points were established for joint angle measurement using both the UG [10] and the Goniwear (Supplementary Table 1).

**Figure 2:**
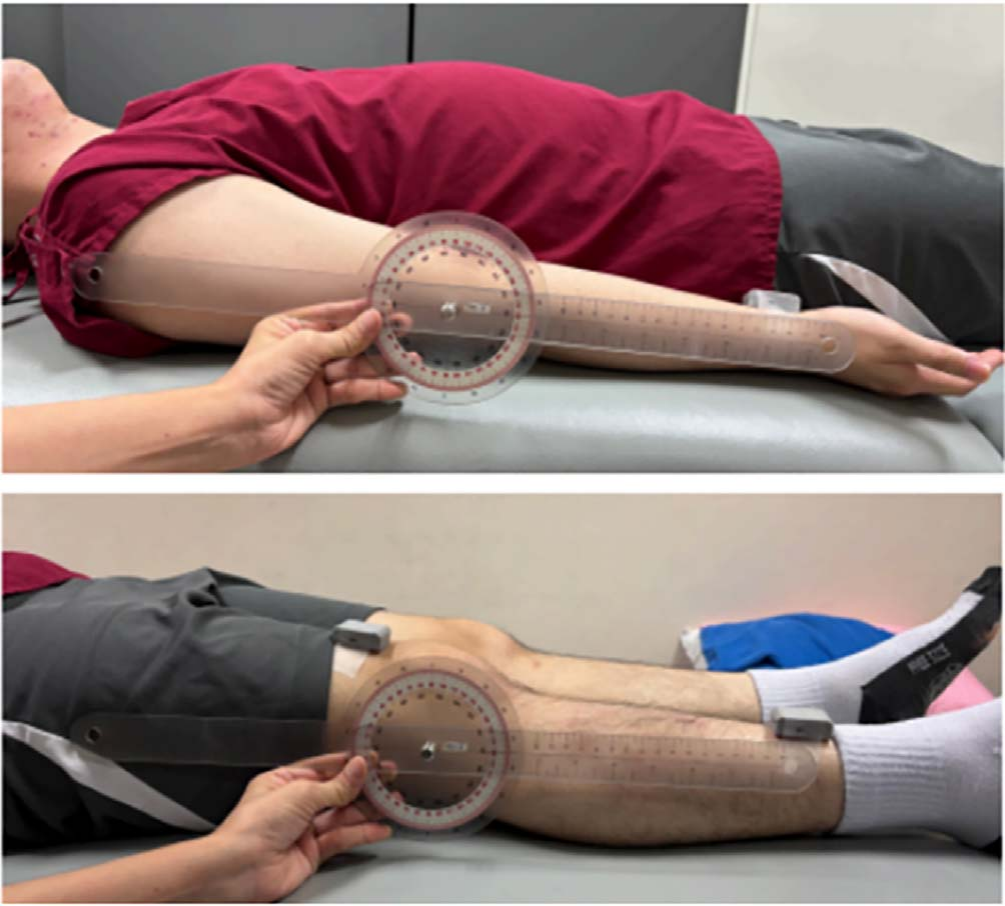
Measuring elbow and knee joints, both the electro-goniometer (Goniwear) and the universal goniometer.

Elbow and knee joint measurements were performed with participants in the supine position. Elbow flexion and extension were assessed with the shoulder maintained in a neutral position. For knee joint assessment, participants remained supine and were instructed to flex the hip and knee simultaneously to achieve maximal knee flexion, followed by knee extension to the neutral position. Joint ROM was assessed using a UG concurrently with real-time angular data acquisition from the Goniwear (Figure 2). Measurements from both instruments were synchronized by recording values at the identical end range of each movement.

In this study, measurements were conducted by a single examiner for the UG and a single examiner for the Goniwear. The UG examiner had undergone validation by an expert physical therapist with more than 20 years of experience in musculoskeletal conditions and demonstrated satisfactory test-retest reliability prior to data collection. The examiner responsible for reading and recording values from the UG was blinded to the Goniowear output, which was not visible during data collection, to minimize observer bias. Data obtained from both instruments were kept confidential until the completion of the experiment. Measurements were performed three times, and the mean value was recorded. Both active and passive movements were evaluated because passive measurements primarily reflect the intrinsic accuracy of the device, whereas active measurements represent dynamic joint movement and functional performance [10].

Participants then performed three repetitions of active movement, followed by three repetitions of passive movement, which was administered by the examiner. Movements were assessed in the following sequence: elbow flexion, elbow extension, knee flexion, and knee extension. To minimize the potential effects of muscular fatigue between trials, a standardized rest interval of approximately 30 s was provided between successive joint movement assessments, in accordance with established biomechanical and musculoskeletal testing protocols.

The Bland-Altman method was used to determine the limits of agreement (LoA) between the UG and Goniwear. The intraclass correlation coefficients (ICC) were calculated using a two-way random-effects model with absolute agreement to assess consistency between the UG and the Goniwear. ICC less than 0.50 can be interpreted as poor, 0.50–0.75 is defined as moderate, 0.75–0.90 is identified as good, and > 0.90 indicates excellent agreement [11].

## Results

Participants were enrolled in two age groups: 20–39 years and 40–59 years, with an equal number of male and female participants in each group. However, due to technical errors during data acquisition, some data were lost and subsequently excluded from the analysis. As a result, a total of 39 participants were included in the final analysis (see Table 1).

**Table 1:** Characteristic data of the participants.

Characteristic	Total	Young adults	Middle-aged adults
Sex			
Male (%)	20 (51.28%)	10 (50.00%)	10 (52.63%)
Female (%)	19 (48.72%)	10 (50.00%)	9 (47.37%)
Age (years)	36.08 ± 15.05	22.20 ± 1.58	50.68 ± 6.03
Weight (km)	60.58 ± 9.05	61.08 ± 10.22	60.05 ± 7.90
Height (cm)	165.21 ± 9.16	167.90 ± 9.31	162.37 ± 8.32
Body mass index (kg/m^2^)	22.10 ± 1.89	21.52 ± 2.00	22.70 ± 1.59
Circumference			
Dominant forearm (cm)	23.40 ± 2.47	22.35 ± 3.13	24.50 ± .00
Dominant arm (cm)	26.99 ± 3.32	26.48 ± 3.54	27.53 ± 3.08
Dominant thigh (cm)	46.27 ± 5.77	46.42 ± 7.02	46.11 ± 4.26
Dominant leg (cm)	34.89 ± 3.07	35.18 ± 3.27	34.58 ± 2.89

**Figure 3:**
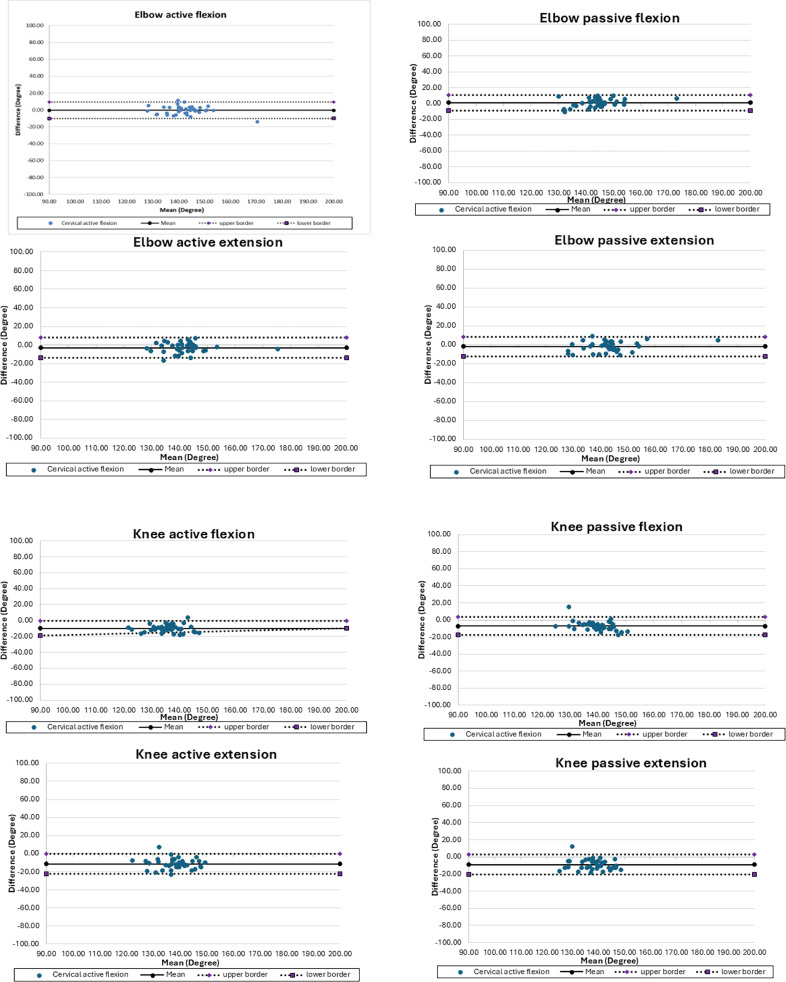
Bland-Altman plot of the range of motion of the elbow and knee joints.

The results demonstrated moderate to good agreement across all measured joint movements. The good agreement was observed in elbow active and passive ROM, while slightly lower ICC values were noted for knee flexion and knee extension. Agreement between the UG and Goniwear was evaluated using ICC, mean differences, and Bland–Altman LoA for both active and passive movements (Table 2, Figure 3).

**Table 2:** Agreement and reliability between the universal goniometer and Goniwear during active and passive movements.

Joint movement	Active movement				Passive movement			
	ICC (2,1)	95%CI	Mean diff	LoA (°)	ICC	95%CI	Mean diff	LoA (°)
Elbow Flexion	0.89	0.80 to 0.95	−0.43	−10.16 to 9.29	0.89	0.78 to 0.94	0.96	−8.84 to 10.87
Elbow Extension	0.84	0.64 to 0.92	−2.97	−13.83 to 7.89	0.91	0.83 to 0.96	−1.71	−12.10 to 8.67
Knee Flexion	0.48	−0.18 to 0.81	−9.97	−19.30 to 0.63	0.55	−0.21 to 0.82	−7.05	−14.68 to 3.57
Knee Extension	0.44	−0.194 to 0.776	−11.16	−22.39 to 0.08	0.46	−0.229 to 0.776	−9.09	−20.74 to 2.55

ICC, intraclass correlation coefficient; CI, confidence interval. LoA, limits of agreement. Note: ICCs were calculated using a two-way random-effects model with absolute agreement [ICC (2,1)]. ICC interpretation followed commonly accepted thresholds: poor (<0.50), moderate (0.50–0.75), good (0.75–0.90), and excellent (>0.90).

Significant associations were observed between limb anthropometric characteristics and joint ROM. The arm circumference and forearm were significantly correlated with elbow joint ROM, and lower limb circumference was significantly correlated with knee joint ROM. In addition, BMI demonstrated a significant relationship with both elbow and knee joint angles. These findings indicate that larger limb girth and higher BMI were associated with alterations in the measured ROM values (Table 3 and Supplementary Table 2).

**Table 3:** Correlation between circumference and joint movement.

	Active movement		Passive movement	
	Knee active flexion *r* (*p*-value	Knee extension *r* (*p*-value	Knee flexion *r* (*p*-value	Knee extension *r* (*p*-value
Thigh circumference	−0.332 (.039)	−0.389 (.012)	−0.349 (.029)	−0.243 (.136)
Leg circumference	−0.622 (<.001)	−0.629 (<.001)	−0.667 (<.001)	−0.529 (<.001)
Body mass index	−0.487 (.002)	−0.486 (.002)	−0.464 (.003)	−0.410 (.010)
	Elbow flexion *r* (*p*-value	Elbow extension *r* (*p*-value	Elbow flexion *r* (*p*-value	Elbow extension *r* (*p*-value
Arm circumference	−0.420 (.008)	−0.481 (.002)	−0.449 (.004)	−0.537 (<.001)
Forearm circumference	−0.415 (.009)	−0.305 (.059)	−0.318 (.048)	−0.496 (.001)
Body mass index	−0.371 (.020)	−0.466 (.003)	−0.387 (.015)	0.419 (.008)

## Discussion

This study aimed to develop an electro goniometer, named Goniwear, displaying joint ROM continuously and in real time in both graphical and numerical formats via a mobile application. The device also allows data storage and retrospective review. The present study demonstrated that high agreement was observed in elbow movements, whereas lower agreement was found in knee movements.

The consistently high ICC values and narrow LoA observed in elbow flexion–extension can be attributed to the biomechanical simplicity of the elbow joint, which functions predominantly as a hinge joint with a well-defined axis of rotation and relatively limited soft-tissue artefact. These findings are consistent with previous studies demonstrating that sensor-based kinematic systems show superior reliability in joints with constrained degrees of freedom and clearly identifiable rotational axes [12–14].

Knee movements demonstrated lower ICC values and systematic negative mean differences, indicating that the Goniwear tended to overestimate joint angles compared with the UG. The knee is a complex joint with combined rolling and sliding mechanics rather than a single fixed axis of rotation, which increases susceptibility to alignment error during angle measurement [15]. In addition, the soft-tissue artefact caused by muscle bulk, adipose tissue, and skin displacement can alter the relative motion between the sensor and the underlying bone [13]. Ancillao et al. [16] found the effect of soft tissue artifact on the knee movement. Consistent with the present study, leg circumference was found to be significantly associated with ROM. Changes in sensor orientation during large-range movements further contribute to measurement variability in wearable systems, particularly near end-range flexion and extension [12, 14]. Therefore, these factors likely explain the reduced agreement. Collectively, these findings suggest that while the Goniwear provides valid and reliable measurements for certain joints and movement planes, caution is warranted when interpreting measurements from joints with complex kinematics or substantial soft tissue motion. Further, the findings indicate that limb circumference and anthropometric indices showed stronger relationships with measurements obtained from the UG compared with the electro-goniometer. Accordingly, clinicians should consider body proportions when interpreting measurements obtained using a UG.

Muscle flexibility and muscle thickness may also indirectly affect IMU measurements through their influence on skin movement artifacts. Variations in muscle tension and soft tissue deformation during joint motion can cause relative displacement between the skin-mounted sensor and the underlying skeletal structure, leading to misalignment between the sensor reference frame and the true joint axis [7, 13]. This phenomenon, commonly referred to as soft tissue artifact, is particularly pronounced in individuals with greater muscle mass or during movements approaching end-range joint positions [17]. Consequently, these factors may contribute to inter-individual variability and should be carefully considered when interpreting IMU-based joint kinematic data in both clinical and research settings. Increased soft tissue mass surrounding the elbow and knee joints may alter sensor positioning and stability, leading to motion artifact and measurement error. In individuals with larger limb girth, the sensor may not remain rigidly coupled to the underlying bone during movement, resulting in relative motion between the skin and the skeletal segment (soft-tissue artefact). This phenomenon is particularly pronounced at the knee joint, where substantial muscle bulk and subcutaneous tissue can shift during flexion and extension, thereby degrading signal fidelity and contributing to lower agreement and wider LoA observed in this study. The findings indicate that limb circumference and anthropometric indices showed stronger relationships with measurements obtained from the UG compared with the Goniwere. Accordingly, clinicians should consider body proportions when interpreting measurements obtained using a UG.

Additionally, higher BMI can impair the identification of anatomical landmarks required for proper device alignment. Misalignment between the Goniwear hinge axis and the true joint rotation axis introduces systematic angular error, while IMU sensors are susceptible to orientation drift and integration error when the sensor reference frame is not consistently maintained. These factors may explain why the elbow joint demonstrated higher reliability and agreement than the knee joint, as the elbow has more prominent bony landmarks and less soft tissue displacement during motion. Further, several biomechanical and subject-specific factors may influence the accuracy of joint angle measurements obtained from IMUs. Movement velocity has been shown to affect IMU signal quality, as rapid or abrupt movements can increase sensor noise, integration error, and signal drift, particularly when gyroscope data are integrated over time [18, 19]. In contrast, slower and more controlled movements generally result in more stable and reliable IMU-derived kinematic data.

Some limitations of this study were not accounted for. Measurements were conducted by a single examiner for the UG and a single examiner for the Goniwear, although formal inter-rater reliability was not assessed. Thus, the intra-rater reliability of the examiners themselves could contribute to the variance observed in the Bland–Altman plots. Future studies should evaluate intra- and inter-rater reliability across multiple examiners. The sample size was determined based on Rasch model methods for scale development rather than power calculations specific to biomechanical outcomes. Although a sample of 40 participants aligns with ranges commonly used in device validation studies, future research should consider biomechanically driven sample size calculations to increase precision in agreement estimates. The UG was used as the reference instrument because it is widely applied in routine clinical assessment of joint ROM. However, it should be acknowledged that the UG is not considered a true gold standard; therefore, the present study evaluates the level of agreement between the electro-goniometer (i.e., Goniwear) and a commonly used clinical measurement tool rather than absolute criterion validity. Furthermore, the findings are specific to the hardware and software versions of the electronic device used and may not generalize to all digital inclinometers or smartphone-based applications. Finally, this study included only healthy participants, which limits generalizability to clinical populations; future research will evaluate the device in patients with musculoskeletal or neurological conditions.

## Conclusion

The Goniwear demonstrates sufficient agreement for use in distal limb joints. However, lower reliability, wider limits of agreement, and significant systematic bias were observed in knee measurements, indicating that traditional goniometry remains the “gold standard” for these joints; thus, knee ROM obtained with the Goniwear should be interpreted with caution. In addition, anthropometric characteristics should be considered a methodological factor influencing measurement validity rather than solely a biological determinant of ROM.

## Data Availability

The original contributions presented in this study are included in the article. Further inquiries can be directed to the corresponding author.

## Supplementary material

*Supplementary Table 1.* Reference points of the Universal goniometer and the Goniwear. *Supplementary Table 2.* Pearson correlation between circumference and body mass index in universal goniometer and Goniwear. The supplementary material of this article is available at https://www.sicot-j.org/10.1051/sicotj/2026016/olm.
