# Supplementary material for: Development and measurement of elbow and knee joints using an electro-goniometer in healthy subjects: A preliminary study
Source: SICOT J. 2026 May 5;12:23. doi: 10.1051/sicotj/2026016 (PMC13143208; doi:10.1051/sicotj/2026016)
Supplement: Supplementary file 1 — Supplementary Table 1. Reference points of the Universal goniometer and the Goniwear. [file sicotj-12-23-s1.pdf]

Supplementary Table 1: Reference points of the Universal goniometer and the Goniwear

Joint	Goniometer	Movement	Stationary arm / Reference sensor (sensor I)	Axis	Moving arm / Moving sensor (sensor II)
elbow	Universal	Flexion	Parallel to the longitudinal axis of the humerus, pointing toward the tip of the acromion process.	The axis is placed over the lateral epicondyle of the humerus	Parallel to the longitudinal axis of the radius, pointing toward the styloid process of the radius.
		Extension	Parallel to the longitudinal axis of the humerus, pointing toward the tip of the acromion process.	The axis is placed over the lateral epicondyle of the humerus	Parallel to the longitudinal axis of the radius, pointing toward the styloid process of the radius.
	Goniwear	Flexion / Extension	Acromion process of the opposite side		distal part of the forearm above the styloid process of the ulna & the styloid process of the radius 3 cm, anterior side
Knee	Universal	Flexion	Parallel to the longitudinal axis	The axis is placed over the lateral	Parallel to the longitudinal axis of the

			of the femur, pointing toward the greater trochanter.	epicondyle of the femur	fibula, pointing toward the lateral malleolus.
		Extension	Parallel to the longitudinal axis of the femur, pointing toward the greater trochanter.	The axis is placed over the lateral epicondyle of the femur	Parallel to the longitudinal axis of the fibula, pointing toward the lateral malleolus.
	Goniwear	Flexion / Extension	8 cm above the midline, anterior side, between the lateral and medial condyles of the femur		5 cm above the midline between the lateral and medial Malleolus
