# Supplementary material for: Development and measurement of elbow and knee joints using an electro-goniometer in healthy subjects: A preliminary study
Source: SICOT J. 2026 May 5;12:23. doi: 10.1051/sicotj/2026016 (PMC13143208; doi:10.1051/sicotj/2026016)
Supplement: Supplementary file 2 — Supplementary Table 2. Pearson correlation between circumference and body mass index in universal goniometer and Goniwear. [file sicotj-12-23-s2.pdf]

Supplementary Table 2 Pearson correlation between circumference and body mass index in universal goniometer and Goniwear

	Universal goniometer			Goniwear		
	Arm circumference r (p-value)	Forearm circumference r (p-value)	Body mass index r (p-value)	Arm circumference r (p-value)	Forearm circumference r (p-value)	Body mass index r (p-value)
Elbow active flexion	-0.421 (.008)	-0.385 (.016)	-0.369 (.021)	-0.380 (.017)	-0.404 (.011)	-0.340 (.034)
Elbow passive flexion	-0.476 (.002)	-0.329 (.041)	-0.426 (.007)	-0.368 (.021)	-0.271 (.095)	-0.298 (.066)
Elbow active extension	-0.455 (.004)	-0.302 (.062)	-0.457 (.003)	-0.451 (.004)	-0.271 (.095)	-0.420 (.008)
Elbow passive extension	-0.516 (<.001)	-0.435 (.006)	-0.418 (.008)	-0.519 (<.001)	-0.528 (<.001)	-0.387 (.015)
	Thigh circumference r (p-value)	Leg circumference r (p-value)	Body mass index r (p-value)	Thigh circumference r (p-value)	Leg circumference r (p-value)	Body mass index r (p-value)
Knee active flexion	-0.382 (.016)	-0.570 (<.001)	-0.414 (.009)	-0.231 (.157)	-0.582 (<.001)	-0.487 (.002)
Knee passive flexion	-0.503 (.001)	-0.722 (<.001)	-0.502 (.001)	-0.191 (.245)	-0.531 (<.001)	-0.371 (.017)
Knee active extension	-0.443 (.005)	-0.625 (<.001)	-0.508 (<.001)	-0.284 (.080)	-0.521 (<.001)	-0.380 (.017)
Knee passive extension	-0.426 (.007)	-0.608 (<.001)	-0.441 (.005)	-0.031 (.849)	-0.351 (.028)	-0.298 (.066)
